# Glucose-Stimulated Calcium Dynamics in Islets of Langerhans in Acute Mouse Pancreas Tissue Slices

**DOI:** 10.1371/journal.pone.0054638

**Published:** 2013-01-24

**Authors:** Andraž Stožer, Jurij Dolenšek, Marjan Slak Rupnik

**Affiliations:** 1 Institute of Physiology, Faculty of Medicine, University of Maribor, Maribor, Slovenia; 2 Centre of Excellence for Integrated Approaches in Chemistry and Biology of Proteins, Ljubljana, Slovenia; University of Bremen, Germany

## Abstract

In endocrine cells within islets of Langerhans calcium ions couple cell stimulation to hormone secretion. Since the advent of modern fluorimetry, numerous *in vitro* studies employing primarily isolated mouse islets have investigated the effects of various secretagogues on cytoplasmic calcium, predominantly in insulin-secreting beta cells. Due to technical limitations, insights of these studies are inherently limited to a rather small subpopulation of outermost cells. The results also seem to depend on various factors, like culture conditions and duration, and are not always easily reconcilable with findings *in vivo*. The main controversies regard the types of calcium oscillations, presence of calcium waves, and the level of synchronized activity. Here, we set out to combine the *in situ* acute mouse pancreas tissue slice preparation with noninvasive fluorescent calcium labeling and subsequent confocal laser scanning microscopy to shed new light on the existing controversies utilizing an innovative approach enabling the characterization of responses in many cells from all layers of islets. Our experiments reproducibly showed stable fast calcium oscillations on a sustained plateau rather than slow oscillations as the predominant type of response in acute tissue slices, and that calcium waves are the mechanistic substrate for synchronization of oscillations. We also found indirect evidence that even a large amplitude calcium signal was not sufficient and that metabolic activation was necessary to ensure cell synchronization upon stimulation with glucose. Our novel method helped resolve existing controversies and showed the potential to help answer important physiological questions, making it one of the methods of choice for the foreseeable future.

## Introduction

Calcium Ca^2+^ ions are likely the most protean cellular secondary messenger, controlling cell survival, gene expression, and coupling excitation with contraction and secretion. Moreover, when cells in a tissue are interconnected through gap junctions, Ca^2+^ signals can spread between them to coordinate the activity of a large number of cells [1]. In endocrine cells in islets of Langerhans, Ca^2+^ is the common denominator for metabolic, neuronal, hormonal, as well as pharmacological influences, regulating exocytosis of islet hormones [2]. Since the introduction of modern fluorimetry as a tool to assess the concentration of free ions in living cells [3], [4], numerous studies employing different experimental models have investigated the effects of different secretagogues on cytoplasmic Ca^2+^ in endocrine cells from islets of Langerhans, predominantly in the most abundant cell type, insulin-secreting beta cells. In these cells, according to the current consensus model, glucose and other nutrients increase production of ATP, thereby decreasing the open probability of the ATP-sensitive potassium (K_ATP_) channels in the plasma membrane. This in turn depolarizes the plasma membrane and leads to an influx of Ca^2+^ through voltage-dependent calcium channels. Ca^2+^ influx has been described to occur rhythmically due to a rhythmic nature of the underlying electrical activity. The exact pattern of changes of intracellular concentration of Ca^2+^ ([Ca^2+^]_i_) upon stimulation depends on the experimental model employed.

In isolated mouse islets, which are currently the most often used experimental model, a considerable overlap has been demonstrated between the glucose-stimulated electrical activity occurring in the form of membrane potential bursts and the [Ca^2+^]_i_ oscillations of comparable frequency, regularity, and durations [5], [6]. Also, oscillations in secreted insulin, driven by oscillations of [Ca^2+^]_i_ have been demonstrated, indicating a very good temporal correlation between electrical activity, Ca^2+^ signaling, and insulin secretion in isolated islets [7], [8]. Moreover, [Ca^2+^]_i_ as well as membrane potential oscillations recorded *in vivo* were very similar to the ones described in isolated islets studied shortly after isolation [5], [6], [9], [10].

In experiments where frequent solution changes and a high perifusion rate are necessary, islets need to be cultured longer to adhere to the cover slips [11]. However, longer culture duration and use of stimulatory glucose concentration during culture seem to be associated with the appearance of oscillations with quite different properties. Whilst in islets *in vivo* and in cultured islets studied within 24 hours after enzyme-assisted isolation, fast [Ca^2+^]_i_ oscillations (frequency = 2–7 min^−1^) that correspond to bursts of membrane potential are the predominant type of response, in islets exposed to stimulatory glucose concentrations during longer culture, slow [Ca^2+^]_i_ oscillations (frequency = 0.2–1 min^−1^) prevail, either as the sole pattern of activity, or with superimposed fast oscillations [5], [6], [8], [12]–[15]. Regarding their temporal characteristics, these slow [Ca^2+^]_i_ oscillations closely resemble slow [Ca^2+^]_i_ oscillations described in dispersed beta cells [14], [16]. During culture, both types of oscillations gradually deteriorate, but the fast ones do so more rapidly [8], [12], [15]. Noteworthy, it has been shown that culture in basal glucose concentration (5.5 mM) minimizes rundown of both types of oscillations, pointing to the possibility that high glucose, in addition to the duration, might contribute to observed changes in [Ca^2+^]_i_ pattern after culture [8]. Many important conclusions about normal beta cell physiology have been drawn from studies with isolated islets, also the ones displaying slow [Ca^2+^]_i_ oscillations, and cultured isolated islets remain the most important experimental model, despite the possibility that the observed [Ca^2+^]_i_ patterns might partially be an experimental artifact.

In experiments in fresh isolated islets as well as in islets *in vivo*, a considerable degree of synchronicity in electrical activity in different cells in the same islet has been shown [5], [9], [17]–[19], along with [Ca^2+^]_i_ oscillations occurring synchronously throughout the islet, with only short phase lags of up to 1–2 seconds detected between oscillations in different islet regions [5], [6], [9]. In islets cultured over longer periods of time, [Ca^2+^]_i_ oscillatory behavior was shown to emerge from waves originating in most sensitive cells and spreading across or alongside periphery of islets [20]–[22]. From time lags between onsets of [Ca^2+^]_i_ oscillations in different parts of an islet or pairs of cells, the speed of progression of [Ca^2+^]_i_ waves has been estimated at 20–200 µms^−1^ [20], [21], [23]. It has been proposed that in islets from normal mice [Ca^2+^]_i_ waves are absent and that they appear after several days of culture, although in many studies in shortly cultured islets, time resolution of [Ca^2+^]_i_ imaging systems did not permit detection of possible rapid [Ca^2+^]_i_ waves [24]. Therefore, it remains to be definitely established whether [Ca^2+^]_i_ waves also exist in fresh islets.

In addition to the confusion regarding types of oscillations present in normal islets and the mechanistic substrate of synchronicity, there is an important technical drawback associated with the use of isolated islets in [Ca^2+^]_i_ imaging experiments. The uptake of fluorescent indicators into the core of isolated islets is limited by diffusion and dye trapping in outermost cells [23], [25]–[27]. Consequently, characterization of responses and a reliable assessment of synchronization in a large number of cells from all layers of an islet have not been possible to date. In addition, by studying only the cells in the perimeter of an islet, one is introducing the bias of selecting the types of cells that are present there.

To shed new light on the controversy regarding types of oscillations present in beta cells, to find out whether calcium waves normally spread across islets and synchronize the activity of beta cells, and to be able to characterize responses in many cells from all layers of islets, we decided to combine the acute pancreas tissue slice technique [28] with live cell [Ca^2+^]_i_ imaging employing confocal laser scanning microscopy (CLSM). In the tissue slice, by virtue of using thin transversal sections, one inherently gains access to cells distributed across the whole cross-sectional area of an islet [29]. Furthermore, the experimental procedure is fast and does not involve any enzymatic steps or cultivation.

Applying this novel approach, we gained access to all layers of cells, characterized their responses, and showed that in islets of Langerhans from acute pancreas tissue slices, fast [Ca^2+^]_i_ oscillations superimposed on a sustained plateau are the predominant type of response and that these oscillations are synchronized via [Ca^2+^]_i_ waves originating in a group of cells and repeatedly spreading across islets. The absence of clear spatial patterns and long time lags between individual cells during activation, despite large amplitudes of [Ca^2+^]_i_ changes, confirm heterogeneity of beta cells and provide evidence that local [Ca^2+^]_i_ increases cannot be efficiently communicated throughout an islet until the majority of cells are metabolically activated. Once the islet as a whole is active, [Ca^2+^]_i_ waves as well as large and rapid [Ca^2+^]_i_ decreases efficiently spread to all cells, producing synchronized oscillatory activity and a synchronous deactivation.

## Materials and Methods

### 1. Ethics Statement

We carried out the study in strict accordance with all national and European recommendations pertaining to work with experimental animals, and all efforts were made to minimize suffering of animals. The protocol was approved by the Veterinary Administration of the Republic of Slovenia (permit number: 34401-61-2009/2).

### 2. Tissue Slice Preparation

We prepared tissue slices from pancreata of 10–20 week old NMRI mice of either sex as described previously [28]. Briefly, we sacrificed the animals by cervical dislocation, accessed the abdomen via laparotomy, and injected low-melting point 1.9% agarose (Lonza Rockland Inc., Rockland, Maine, USA) in extracellular solution (ECS, consisting of (in mM) 125 NaCl, 26 NaHCO_3_, 6 glucose, 6 lactic acid, 3 myo-inositol, 2.5 KCl, 2 Na-pyruvate, 2 CaCl_2_, 1.25 NaH_2_PO_4_, 1 MgCl_2_, 0.5 ascorbic acid) at 40°C into the proximal common bile duct clamped distally at the major duodenal papilla. After injection, the pancreas was cooled with an ice-cold ECS. The agarose-permeated pancreas was then extracted and gently washed in ice-cold ECS. Small blocks of tissue (0.1–0.2 cm^3^ in size) were cut from the organ, cleared of connective tissue, and transferred to a 5 ml Petri dish filled with agarose at 40°C and immediately cooled on ice. Individual cubes containing tissue blocks were cut from hardened agarose and glued (Super Attak, Henkel Slovenija d.o.o., Maribor, Slovenia) onto the sample plate of the VT 1000 S vibratome (Leica, Nussloch, Germany). The tissue was cut at 0.05 mm s^−1^ at 70 Hz into 140 µm-thick slices of a surface area of 20–100 mm^2^. Throughout preparation and during slicing we held the tissue in an ice-cold extracellular solution (ECS, consisting of (in mM) 125 NaCl, 26 NaHCO_3_, 6 glucose, 6 lactic acid, 3 myo-inositol, 2.5 KCl, 2 Na-pyruvate, 2 CaCl_2_, 1.25 NaH_2_PO_4_, 1 MgCl_2_, 0.5 ascorbic acid) continuously bubbled with a gas mixture containing 95% O_2_ and 5% CO_2_ at barometric pressure to ensure oxygenation and a pH of 7.4. After cutting slices were collected in 30 ml of HEPES-buffered saline at room temperature (HBS, consisting of (in mM) 150 NaCl, 10 HEPES, 6 glucose, 5 KCl, 2 CaCl_2_, 1 MgCl_2_; titrated to pH = 7.4 using 1 M NaOH) before they were incubated in the dye-loading solution. All chemicals were obtained from Sigma-Aldrich (St. Louis, Missouri, USA) unless otherwise specified.

### 3. Dye Loading

Up to 10 slices were incubated in a 5 ml Petri dish, exposed to ambient air but protected from light, and filled with 3.333 ml of HBS containing 6 µM Oregon Green 488 BAPTA-1 AM calcium dye (OGB-1, Invitrogen, Eugene, Oregon, USA), 0.03% Pluronic F-127 (w/v), and 0.12% dimethylsulphoxide (v/v) for 50 minutes on an orbital shaker (40 turns min^−1^) at room temperature. OGB-1 uptake was limited to the first two or three most superficial cell layers as described previously for pituitary slices [30], [31] and isolated islets [23], [27]. Several-fold differences in fluorescence intensity were observed between cells, most probably due to differences in viability, enzyme activity, loading, and variable extrusion of the dye. Different loading did not influence fluorescence time profiles. After staining and before measurements, the slices were kept protected from ambient light in 30 ml of fresh HBS for up to 12 hours at room temperature. HBS was exchanged every 2 hours. Individual slices were transferred to a temperature-controlled bath chamber (37°C, Luigs & Neumann, Ratingen, Germany) continuously superfused with bubbled (5% CO_2_, 95% O_2_) ECS and used in imaging experiments. The slices were held on the bottom of the chamber by a nylon-fibre net spread across a U-shaped platinum-wire weight. The preparation of tissue slices typically lasted up to 4 hours. Throughout the whole preparatory phase, slices were kept in 6 mM glucose.

### 4. Calcium Imaging

Imaging was performed on a Leica TCS SP5 AOBS Tandem II upright confocal system using a Leica HCX APO L water immersion objective (20x, NA 1.0). OGB-1 was excited by an argon 488 nm laser and fluorescence detected by Leica HyD hybrid detector in the range of 500–650 nm (all from Leica Microsystems GmbH, Wetzlar, Germany). 8-bit 512×512 pixels images were acquired every 2 seconds. This frequency has been used previously and reliably resolved [Ca^2+^]_i_ dynamics upon stimulation with various concentrations of glucose [26]. To avoid recording from cells at the potentially damaged cut surface, cells at 15 µm depth or more were imaged. Optical section thickness was 4 µm. Both the thickness of the optical section and the acquisition frequency gave a reasonable trade-off between a satisfactory signal strength at lowest acceptable laser power (to avoid photobleaching and prolong the maximum time of recording) on one hand and the need to keep the optical section thickness as thin as possible to assure recording from a single cell only. Before and after recording time series, a high spatial resolution fluorescence image (1024×1024 pixels) was taken and used as a reference to assess motion artefacts and regions of interest (ROIs) during analysis. High-speed [Ca^2+^]_i_ imaging was employed whenever higher temporal resolution was required. To this end, 8-bit 256×64 pixels images were acquired every 50 milliseconds and 512×128 pixels images were taken and used as a reference.

### 5. Data Analyses and Presentation

Calcium kinetics were measured off-line from ROIs and exported employing Leica Application Suite Advanced Fluorescence software (Leica Microsystems GmbH, Wetzlar, Germany). Further analysis was performed using custom-made scripts in MATLAB program (The MathWorks, Inc., Massachusetts, USA). Photobleaching was accounted for by a combination of linear and exponential fit. Traces were rejected if extensive motion artefacts were observed. The fluorescence signals of OGB-1 were expressed as F/F_0_ ratios, F_0_ representing the initial level of fluorescence and F the fluorescence signal recorded at individual time points during the experiment, respectively. Figures were plotted using the SigmaPlot for Windows Version 11.0 program (Systat Software, Inc., Illinois, USA).

## Results

### 1. Types of Responses to Glucose

As described before [28], low melting point agarose supported the pancreatic tissue from the inside by filling its ductal tree and in addition from the outside, mechanically stabilizing the tissue of the slice. Such an approach made pancreatic tissue suitable for isolation, slicing, transferring, shaking during dye-loading, and for subsequent long-term [Ca^2+^]_i_ imaging. Enzymes from the exocrine part did not appear to have any significant effect upon viability and did not significantly interfere with dye loading. Therefore no enzyme inhibitors were needed. At least partially this can be ascribed to the subphysiological temperatures used throughout isolation, cutting, and dye-loading, to lack of stimuli to activate the enzymes, and to dilution due to large and constant perifusion volumes during experimentation at physiological temperatures. Notably, due to its porous structure, agarose did not importantly impede the exchange of extracellular solution and the access of dye molecules. We checked cell viability and morphology using the LIVE/DEAD® Viability/Cytotoxicity Kit (Sigma-Aldrich, St. Louis, Missouri, USA) [32] and the lipophilic dye di-4 ANNEPS. The endocrine cells survived in the tissue slices throughout the whole duration of the experiment and the characteristic polyhedral shapes, reported previously in isolated islets [33], were well preserved in our preparation (Figure S1, Figure S2, and Video S3).

**Figure 1:**
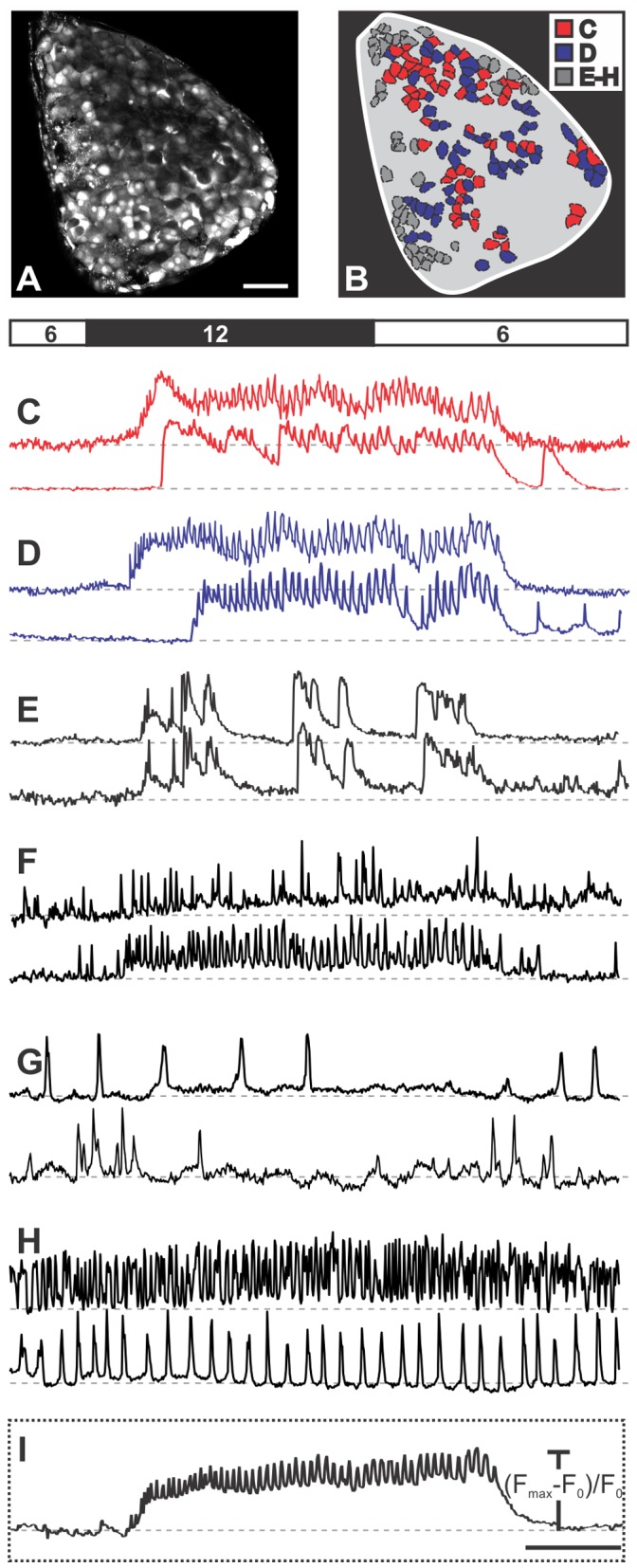
Spatiotemporal [Ca^2+^]_i_ patterns in a representative islet of Langerhans upon stimulation with 12 mM glucose. A A high resolution image, used as a reference to choose regions of interest corresponding to individual cells and to assess motion artefacts, showing that Oregon Green® 488 BAPTA-1 effectively labeled most of the cells within the focal plane. Scale bar indicates 50 micrometers. **B** A schematic color-coded representation of the position of cells in A that responded to stimulation with 12 mM glucose as shown in C–E (N = 177 cells). The grey area indicates unlabelled or unresponsive cells. We detected six different types of responses to glucose. The types of responses presented in C and D were predominant and are characteristic of beta cells. **C** Slow transient response followed by oscillations superimposed on a sustained plateau (type 1, N = 63 cells). Note the difference in time required for activation between the upper and lower trace, the synchronicity of Ca^2+^ oscillations superimposed on the sustained plateau and of deactivation, as well as the presence of a transient increase in Ca^2+^ in the lower trace after the sustained plateau has subsided. **D** Response as in C but without a clear transient phase (type 2, N = 61 cells). **E–H** The responses representative of non typical beta cells and non beta cells (see text for further details). **I** The average time course of fluorescence over the whole islet. Due to synchronicity of Ca^2+^ oscillations in individual beta cells, the oscillations are clearly distinguishable in the average signal. Scale bar indicates 200 seconds. On Y-axes, values represent normalized fraction of the difference between maximum and basal fluorescence.

We took advantage of discriminatory functional differences reported in previous studies [25]–[27], [34] to identify cells in islets of Langerhans from mouse pancreas tissue slices and characterize their responses. At the beginning of recordings, beta cells were exposed to ECS containing 6 mM glucose, the same concentration that we also used throughout the preparatory phase. The islets were then stimulated for 10–30 minutes with 12 mM glucose. Figure 1 features a representative islet encompassing the whole repertoire of types of responses and their spatial distribution within a single islet of Langerhans.

The majority of cells located in the core of the islet displayed no oscillations in 6 mM glucose, responded to 12 mM glucose with oscillations superimposed on an elevated [Ca^2+^]_i_ level and returned to the baseline [Ca^2+^]_i_ upon removal of stimulatory glucose (130 of 177 cells, 73%). Among the latter, approximately a half responded with a transient increase in [Ca^2+^]_i_ followed by a sustained plateau at a level higher than the baseline and lower than the peak of the transient, with [Ca^2+^]_i_ oscillations superimposed on the plateau (type 1, 63 cells, 36%, Figure 1C). In the other half, a clear transient rise above the level of the sustained plateau was missing. However, the [Ca^2+^]_i_ oscillations superimposed on the plateau were clearly in phase with [Ca^2+^]_i_ oscillations in cells of type 1 (type 2, 61 cells, 34%, Figure 1D). In addition, a few cells displayed a third type of response with [Ca^2+^]_i_ intermittently returning to the baseline during continuous stimulation with glucose (type 3, 6 cells, 3%, Figure 1E). Due to the virtual absence of [Ca^2+^]_i_ oscillations in these cells, the level of synchronicity with cells of the former two types could not be properly assessed. Nevertheless, between individual cells of type 3, there was a high degree of synchronicity, discernible by [Ca^2+^]_i_ rising from and returning back to the baseline practically simultaneously in different cells of this type. In all cells of types 1–3 [Ca^2+^]_i_ returned to basal levels upon lowering the glucose back to 6 mM. Occasionally, a single or a few transient increases with superimposed [Ca^2+^]_i_ oscillations, and lasting for up to 100 seconds occurred in some cells of types 1 and 2 after stimulation had subsided and [Ca^2+^]_i_ had returned to the baseline level (lower panels in Figures 1C and 1D).

In the periphery of the islet, cells were detected that displayed oscillations in 6 mM glucose. In the largest subset of these cells, the frequency of oscillations increased upon stimulation with 12 mM glucose. This increase occurred on the baseline or on a slightly elevated [Ca^2+^]_i_ level and was sometimes accompanied by an increase in amplitudes of [Ca^2+^]_i_ oscillations (type 4, 30 cells, 17%, Figure 1F). In another subset of cells the frequency of [Ca^2+^]_i_ oscillations decreased upon exposure to 12 mM glucose or the [Ca^2+^]_i_ oscillations disappeared altogether (type 5, 6 cells, 3%, Figure 1G). In cells of types 4 and 5, the effect of glucose was reversible. In the remaining cells that displayed oscillations in 6 mM glucose, neither the frequency nor the amplitude of [Ca^2+^]_i_ oscillations changed with changing glucose concentration (type 6, 12 cells, 7%, Figure 1H). [Ca^2+^]_i_ oscillations in cells of types 4–6 were not synchronized between homotypic cells. Additionally, except for types 1 and 2, [Ca^2+^]_i_ oscillations were never synchronous in heterotypic cells. Types of responses are summarized in Table 1.

**Table 1:** Summary of types of responses to stimulation with 12 mM glucose.

Type of response	[Ca^2+^]_i_ oscillations in 6 mM glucose	[Ca^2+^]_i_ oscillations in 12 mM glucose	Transient increase of [Ca^2+^]_i_ in 12 mM glucose	Synchronous [Ca^2+^]_i_ oscillations on plateau	Number of cells (out of 178)	%	Location within theislet	Reference to Figure 1
1	–	+	+	+	63	35	Central	1C
2	–	+	–	+	61	34	Central	1D
3	–	+	+	–	6	3	Central	1E
4	+	++	–	–	30	17	Peripheral	1F
5	+	–	–	–	6	3	Peripheral	1G
6	+	+	–	–	12	7	Peripheral	1H

(+) and (−) indicate presence and absence of the specified phenomenon. (++) indicates an increase in frequency.

Noticeably, the remaining cells in the investigated cross-sectional area (indicated by the grey area in Figure 1B) showed no detectable [Ca^2+^]_i_ changes during the applied stimulation protocol. Results in Figure 1 are representative of 17 islets from 10 animals analyzed in this study. In the following, we provide a detailed quantitative analysis of cells of types 1 and 2 that were functionally recognized as typical beta cells. First, we focus on the activation after exposure to stimulatory glucose, then we quantify the behavior during the sustained plateau phase with superimposed [Ca^2+^]_i_ oscillations, and finally we analyze the deactivation process.

### 2. Activation of Beta Cells

**Figure 2:**
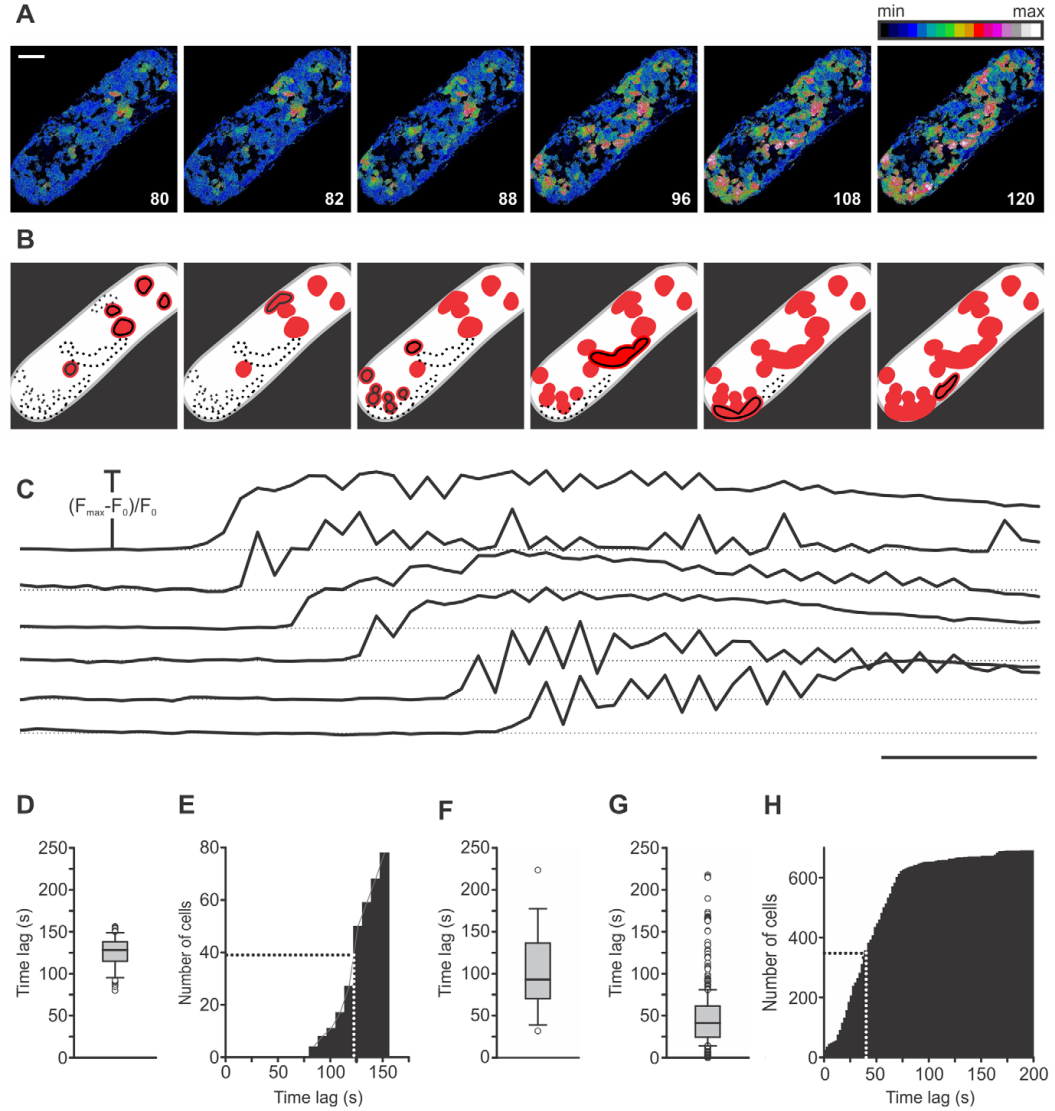
Spatiotemporal characterization of activation of beta cells. A Activation of beta cells. Images are presented as F/F_0_ and pseudocolored with blue and white representing low and high intensity signals, respectively. Scale bar indicates 50 µm. Numbers indicate time after beginning of stimulation in seconds. **B** Regions that activated at a certain time are indicated as black-bordered red areas and regions already activated with red only. Differences between individual cells inside these regions were not resolvable at the recording rate of 0.5 Hz. No regular spatial pattern of activation is visible. **C** Time traces for the regions indicated in B. Scale bar indicates 20 seconds. On Y-axes, values represent normalized fraction of the difference between maximum and basal fluorescence. **D** Distribution of time delays from the beginning of stimulation to activation for 78 cells from this islet (median = 128 s, 1^st^ quartile = 115 s, 3^rd^ quartile = 138 s). **E** Cumulative distribution of time delays from the beginning of stimulation to activation in this islet. First regions responded 80 seconds after the rise in glucose. Within the following 76 seconds all other groups of cells activated. A half of cells activated within 42 seconds after the activation of the first cell. **F** Distribution of time delays from the beginning of stimulation to activation of the first cell in each islet for 17 different islets (median = 93 s, 1^st^ quartile = 72 s, 3^rd^ quartile = 135 s). **G** Distribution of time delays from activation of the first cell in each islet to activation of any given cell from the same islet for 700 cells from 17 different islets (median = 41 s, 1^st^ quartile = 24 s, 3^rd^ quartile = 62 s). **H** Cumulative distribution of time delays shown in G. A half of cells activated within 40 seconds after the activation of the first cell. All cells activated within 312 seconds after the first.

The activation of beta cells, characterized by onsets of [Ca^2+^]_i_ increases, varied greatly in time and space. Figure 2 and Video S1 feature a representative islet with cells responding in groups in which responses of individual cells were not further resolvable at the sampling rate of 0.5 Hz. The responsive groups of cells were physically separated from each other and located in several different regions of the islet. Subsequently activated regions did not follow any clearly discernible spatial rule as they were located to different sides of the first-responders and at variable distances from them. In the particular islet in Figure 2, 80 seconds after the solution containing 12 mM glucose had reached the bath chamber, first cells responded to the stimulus (Figure 2D and 2E). All following groups of cells responded within 76 seconds after the first, with a half time of 42 seconds (Figure 2E). Further analysis on 700 cells pooled from 17 islets showed that the median delay from the beginning of stimulation to activation of the first cell in each islet was 93 seconds (1^st^ quartile = 72 s, 3^rd^ quartile = 135 s, Figure 2F) and that the median delay from activation of the first cell in each islet to activation of any given responsive cell from the same islet was 41 seconds (1^st^ quartile = 24 s, 3^rd^ quartile = 62 s, Figure 2G). The cumulative distribution of the pooled data reveals that a half of all cells responded within 40 seconds (Figure 2H). The cell group that responded last displayed a time delay of 312 seconds after the first (Figure 2G).

### 3. Plateau Phase

**Figure 3:**
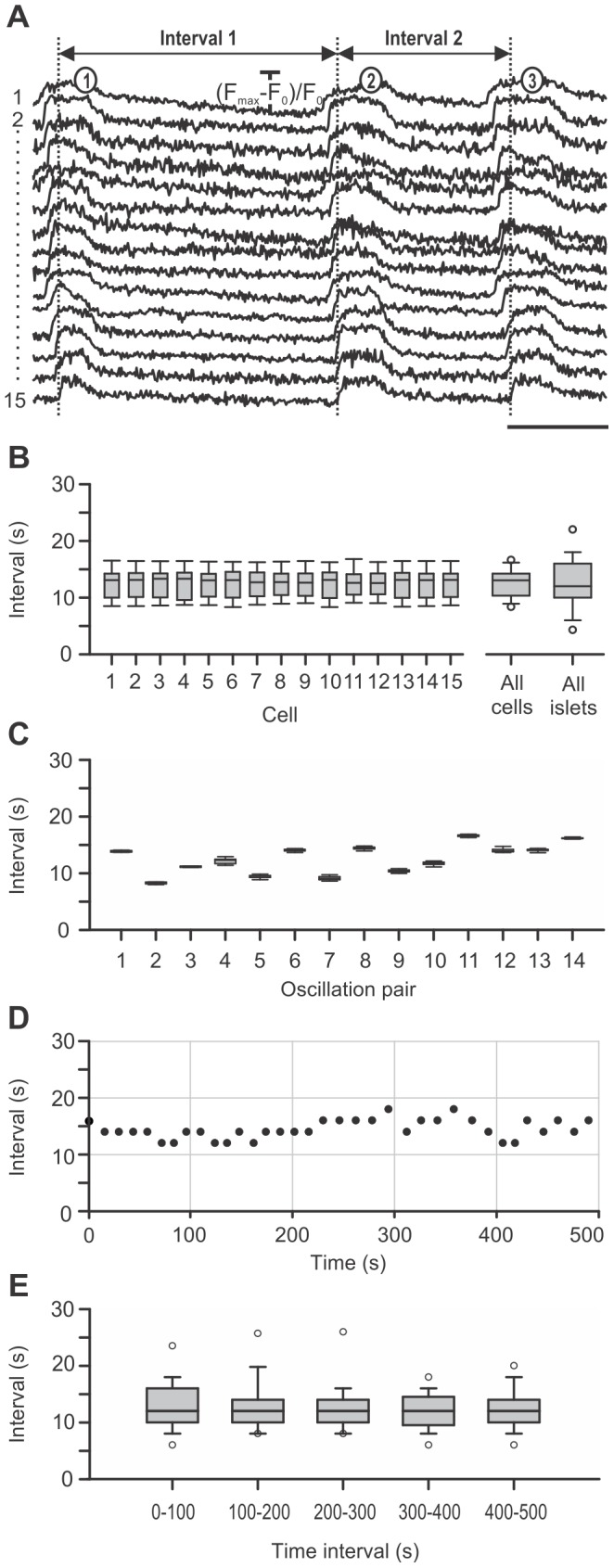
Characterization of frequencies of [Ca^2+^]_i_ oscillations superimposed on the sustained plateau. A Representative traces of 3 subsequent [Ca^2+^]_i_ oscillations in 15 cells analyzed in B and C. Scale bar represents 5 seconds. On Y-axes, values represent normalized fraction of the difference between maximum and plateau baseline fluorescence. **B** Distribution of durations of intervals between consecutive [Ca^2+^]_i_ oscillations for 14 subsequent pairs of [Ca^2+^]_i_ oscillations in 15 cells from a single islet, presented for individual cells. In every cell, roughly the same range of interval durations is present. The box plot indicated with “All cells” shows the distribution of interval durations pooled for 14 subsequent pairs of [Ca^2+^]_i_ oscillations in 15 cells from the same islet (median = 13 s, 1^st^ quartile = 10 s, 3^rd^ quartile = 14 s). The box plot indicated with “All islets” shows the distribution of intervals for all pairs of [Ca^2+^]_i_ oscillations in 12 different islets (median = 12 s, 1^st^ quartile = 10 s, 3^rd^ quartile = 16 s). **C** Distribution of interval durations for 14 subsequent pairs of [Ca^2+^]_i_ oscillations in 15 cells from a single islet, presented for every pair of consecutive [Ca^2+^]_i_ oscillations. Note that intervals change from oscillation to oscillation in roughly the same way in every analyzed cell. **D** Time-variability of the interval durations in a single islet of Langerhans over a period of 500 seconds. Note the absence of any clear trend towards higher or lower frequencies with time. **E** Time-variability of the interval in 12 islets of Langerhans over a period of 500 seconds, shown as the distribution of intervals between [Ca^2+^]_i_ oscillations for five subsequent 100-seconds-long time intervals. For each of the 12 different islets median interval durations were calculated for each of the 100-seconds-long time intervals and compared with each other by employing nonparametric Friedmańs analysis of variance. The duration of intervals between two consecutive [Ca^2+^]_i_ oscillations did not significantly change over the five time intervals.

During the sustained plateau, beta cells displayed [Ca^2+^]_i_ oscillations that were well synchronized between all homotypic cells in an islet, and clearly detectable in the average signal (Figures 1I and 3A). The frequency of [Ca^2+^]_i_ oscillations was assessed by pooling data from 12 different islets displaying regular oscillations upon stimulation with 12 mM glucose and calculating the running peak-to-peak interval over the whole period of oscillations. The median interval was 12 seconds (1^st^ quartile = 10 s, 3^rd^ quartile = 16 s, Figure 3B, “All islets”). To gain a more precise insight into the temporal variability of frequency, we employed high-speed imaging of [Ca^2+^]_i_ oscillations. Figure 3B shows the distribution of intervals between 15 subsequently occurring [Ca^2+^]_i_ oscillations in 15 cells from a single islet of Langerhans. Evidently, in every cell, almost the same range of intervals is present. The degree of variability of the interval between a given pair of [Ca^2+^]_i_ oscillations across different cells within the same islet is shown in Figure 3C. From one [Ca^2+^]_i_ oscillation to another, the interval changed, but these changes were reflected in practically the same way in all 15 cells assessed. Moreover, no clear trend towards higher or lower frequencies with time could be detected in this analysis. To further corroborate the latter finding, we tracked the durations of intervals over a longer time period (500 seconds). In Figure 3D, time-variability of the interval between two consecutive [Ca^2+^]_i_ oscillations is shown for a single representative islet, demonstrating the absence of any clear trend towards changing frequencies even over a longer time period. To quantify the constancy of frequency, for 12 different islets median interval durations were calculated for five 100-seconds-long time intervals and compared with each other [35]. The duration of intervals between two consecutive [Ca^2+^]_i_ oscillations did not significantly change over the five time intervals (Figure 3E).

**Figure 4:**
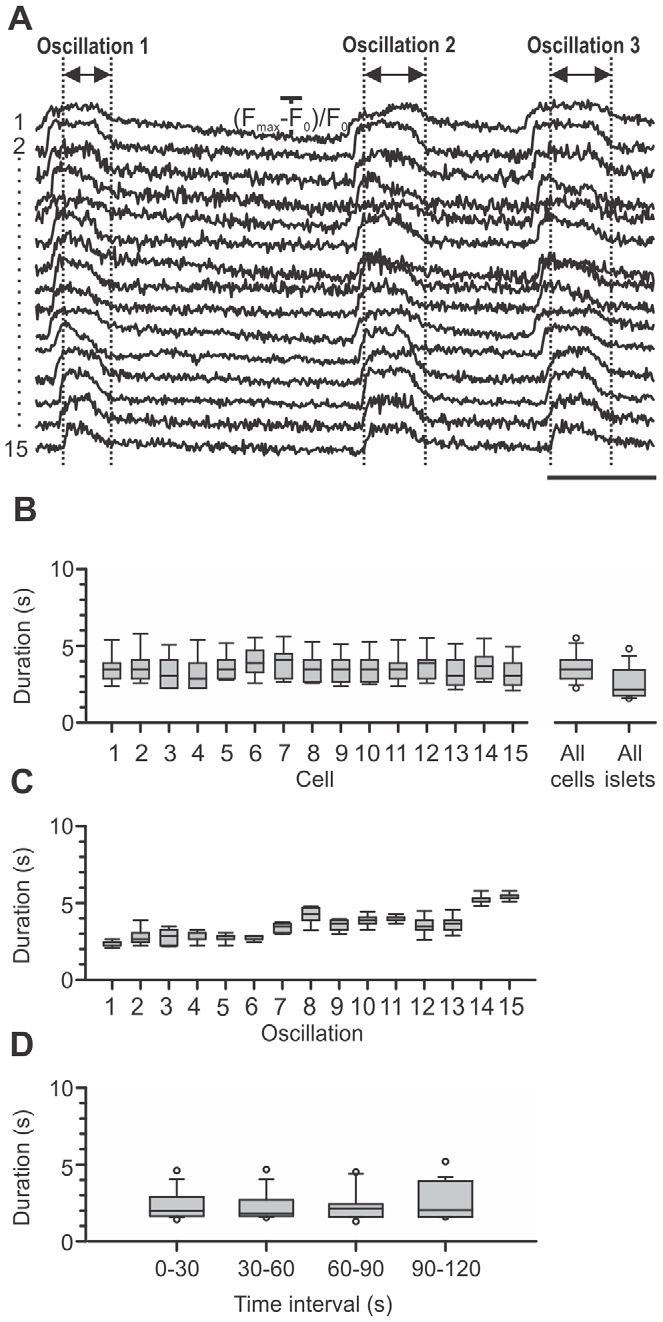
Characterization of durations of [Ca^2+^]_i_ oscillations superimposed on the sustained plateau. A Representative traces of 3 subsequent [Ca^2+^]_i_ oscillations in 15 cells analyzed in B and C. Scale bar represents 5 seconds. On Y-axes, values represent normalized fraction of the difference between maximum and plateau baseline fluorescence. **B** Distribution of durations of Ca^2+^ oscillations for 15 subsequent [Ca^2+^]_i_ oscillations in 15 cells from a single islet presented for individual cells. Note that in every cell, roughly the same range of interval durations is present. The box plot indicated with “All cells” shows the distribution of interval durations pooled for 15 subsequent [Ca^2+^]_i_ oscillations in 15 cells from the same islet (median = 3.5 s, 1^st^ quartile = 2.9 s, 3^rd^ quartile = 4.1 s). The box plot indicated with “All islets” shows the distribution of durations for all [Ca^2+^]_i_ oscillations in 6 different islets (median = 2.2 s, 1^st^ quartile = 1.8 s, 3^rd^ quartile = 3.4 s). **C** Distribution of durations of [Ca^2+^]_i_ oscillations for 15 subsequent [Ca^2+^]_i_ oscillations in 15 cells from a single islet, presented for every individual [Ca^2+^]_i_ oscillation. Note that the durations vary from one oscillation to another in roughly the same way in all analyzed cells. **D** Time-variability of the duration of [Ca^2+^]_i_ oscillations in 6 islets of Langerhans over a period of 120 seconds, shown as the distribution of durations of [Ca^2+^]_i_ oscillations in four subsequent 30-seconds-long time intervals. For each of the 6 different islets median durations of [Ca^2+^]_i_ oscillations were calculated for each of the 30-seconds-long time intervals and compared with each other by employing nonparametric Friedmańs analysis of variance. The duration of [Ca^2+^]_i_ oscillations did not significantly change over the four time intervals.

High speed [Ca^2+^]_i_ imaging also enabled a detailed assessment of durations of [Ca^2+^]_i_ oscillations (Figure 4A). Figure 4B shows the distribution of durations of individual oscillations for 15 subsequent oscillations in 15 cells from the same islet of Langerhans as in Figure 3. A comparison with Figure 4C and with Figures 3B and 3C reveals a similar relationship between intra- and intercellular variability in durations of [Ca^2+^]_i_ oscillations as is the case with intervals between oscillations. Namely, the intracellular variability is comparable between individual cells and larger than the intercellular variability. In 6 islets, the median duration of [Ca^2+^]_i_ oscillations was 2.2 seconds (1^st^ quartile = 1.8 s, 3^rd^ quartile = 3.4 s, Figure 4B, “All islets”). In Figure 4C, a trend towards longer durations with time is visible. To analyze this finding more precisely, we tracked the durations of oscillations over a period of 120 seconds. In Figure 4D, the distributions of durations of oscillations are shown for 6 islets over a period of 120 seconds, divided into four 30-seconds-long time intervals. Median durations of oscillations were calculated for each of the 30-seconds-long time intervals for 6 different islets and compared with each other [35]. The duration of oscillations did not significantly change over the four time intervals. At present, an analysis over a longer time period was not possible due to shorter recording times needed to avoid significant photobleaching.

### 4. Calcium Waves during Plateau Phase

**Figure 5:**
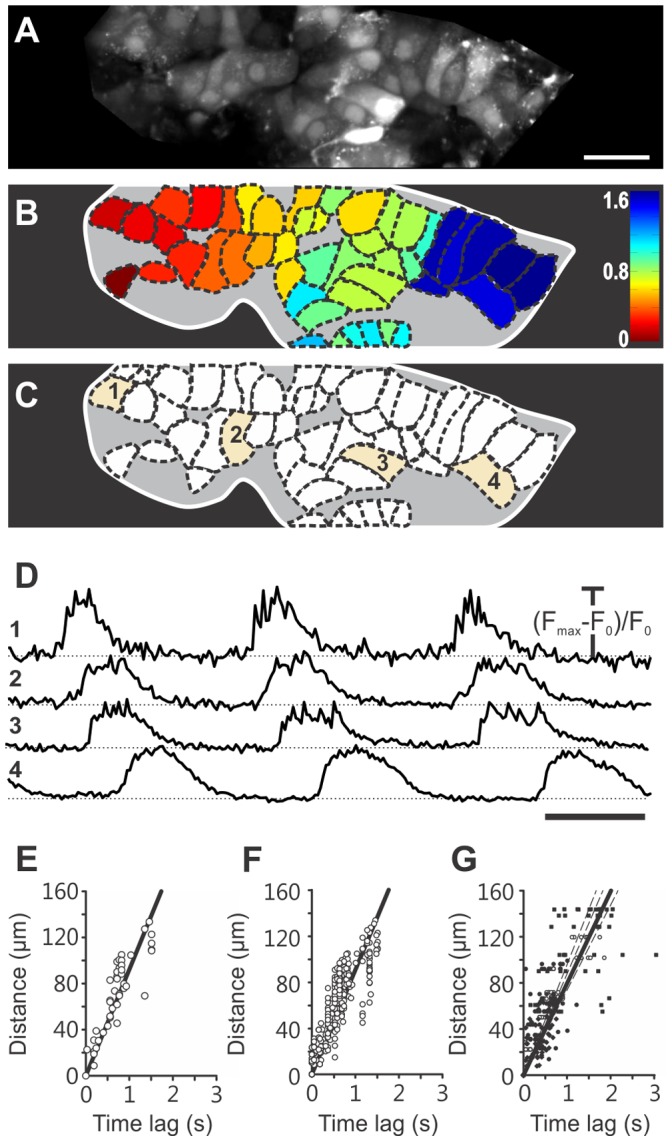
Spatiotemporal characterization of [Ca^2+^]_i_ oscillations superimposed on the sustained plateau. A High resolution image (1024×256 pixels) that served as a reference to select regions of interest indicated in B and to assess possible motion artefacts. Scale bar indicates 20 micrometers. **B** Color-coded time delays for every cell demonstrating the average direction of spreading of [Ca^2+^]_i_ waves for 6 consecutive [Ca^2+^]_i_ oscillations. **C** 45 individual cells whose signals were included in the analyses. Temporal traces of highlighted cells indicated with 1–4 are plotted in D. **D** 3 consecutive [Ca^2+^]_i_ oscillations in 4 cells indicated in C. Scale bar indicates 2 seconds. On Y-axes, values represent normalized fraction of the difference between maximum and plateau baseline fluorescence. **E** Time delays between the beginning of a [Ca^2+^]_i_ oscillation in the cell in which the wave originated and the beginning of the [Ca^2+^]_i_ oscillation in any given cell as a function of the Euclidean distance between the cell of wave origin and the respective cell, for a single [Ca^2+^]_i_ oscillation in 45 cells from the islet shown in A-C. The regression line gives an average velocity of 92 µm s^−1^ (R^2^ = 0.76, p<0.001). **F** After taking into account 6 consecutive spikes in the same set of cells, the same average speed was obtained (R^2^ = 0.73, p<0.001). **G** For 4 different islets, with 6 consecutive [Ca^2+^]_i_ oscillations in 10 cells from each islet, the average speed was 80 µm s^−1^ (R^2^ = 0.62, p<0.001). The respective values obtained in 4 islets were 98 µm s^−1^ (R^2^ = 0.83, p<0.001), 88 µm s^−1^ (R^2^ = 0.34, p<0.001), 80 µm s^−1^ (R^2^ = 0.40, p<0.001), and 74 µm s^−1^ (R^2^ = 0.46, p<0.001). In Figures E-G, x and y axes are chosen as to enable representation of speed by the slope of the regression lines. However, velocities were calculated with distances representing the independent and time lags being the dependent variable.

To quantify the level of synchrony between individual beta cells and estimate the differences in onsets of [Ca^2+^]_i_ oscillations, we made use of high-speed [Ca^2+^]_i_ imaging during an already established sustained plateau with superimposed [Ca^2+^]_i_ oscillations. This way we were able to detect [Ca^2+^]_i_ waves that spread repeatedly across islets in roughly the same direction over and over again. Figure 5 summarizes the results of a typical high-speed [Ca^2+^]_i_ imaging experiment. In Figure 5A, a high spatial resolution image is shown that enabled selection of regions of interest corresponding to individual cells and the assessment of motion artifacts. In order to visualize the direction of [Ca^2+^]_i_ waves, we calculated for every [Ca^2+^]_i_ oscillation the time delay between the beginning of oscillation in the cell that produced the first oscillation and the beginning of the oscillation in any given responsive cell.

To every cell, a value of the median delay was assigned and color-coded as indicated in Figure 5B. Areas of the islet with the lowest delays represent earliest increases in [Ca^2+^]_i_ and thus indicate where the wave originated in the focal plane and vice versa, areas of the islet with the greatest delays represent areas where the wave terminated. For the representative islet in Figure 5, the median value of time delays was obtained from 6 consecutive [Ca^2+^]_i_ oscillations in every cell. In this islet, [Ca^2+^]_i_ waves spread from left to right. This finding is further supported by the time traces in Figure 5D showing the order of appearance of three consecutive [Ca^2+^]_i_ oscillations in 4 cells positioned from left to right as indicated in Figure 5C, as well as by Video S2 that shows 120 seconds of oscillatory activity for this islet. Finally, we estimated the speed of [Ca^2+^]_i_ wave propagation by plotting the time delay between the beginning of a [Ca^2+^]_i_ oscillation in the cell in which the wave originated and the beginning of the oscillation in any given cell as a function of the Euclidean distance between the cell of wave origin and the respective cell and fitting a regression line through the data points. In Figure 5E data points obtained this way for a single [Ca^2+^]_i_ oscillation in 45 cells from the islet shown in Figures 5A–C are plotted along with a regression line yielding an average velocity of 92 µm s^−1^ (R = 0.87, p<0.001). After taking into account 6 consecutive spikes in the same set of cells, the same average speed was obtained (R = 0.85, p<0.001, Figure 5E2). For 4 different islets, with 6 consecutive [Ca^2+^]_i_ oscillations in 10 cells from each islet, the average speed was 80 µm s^−1^ (R^2^ = 0.62, p<0.001, Figure 5E3).

### 5. Deactivation of Beta Cells

**Figure 6:**
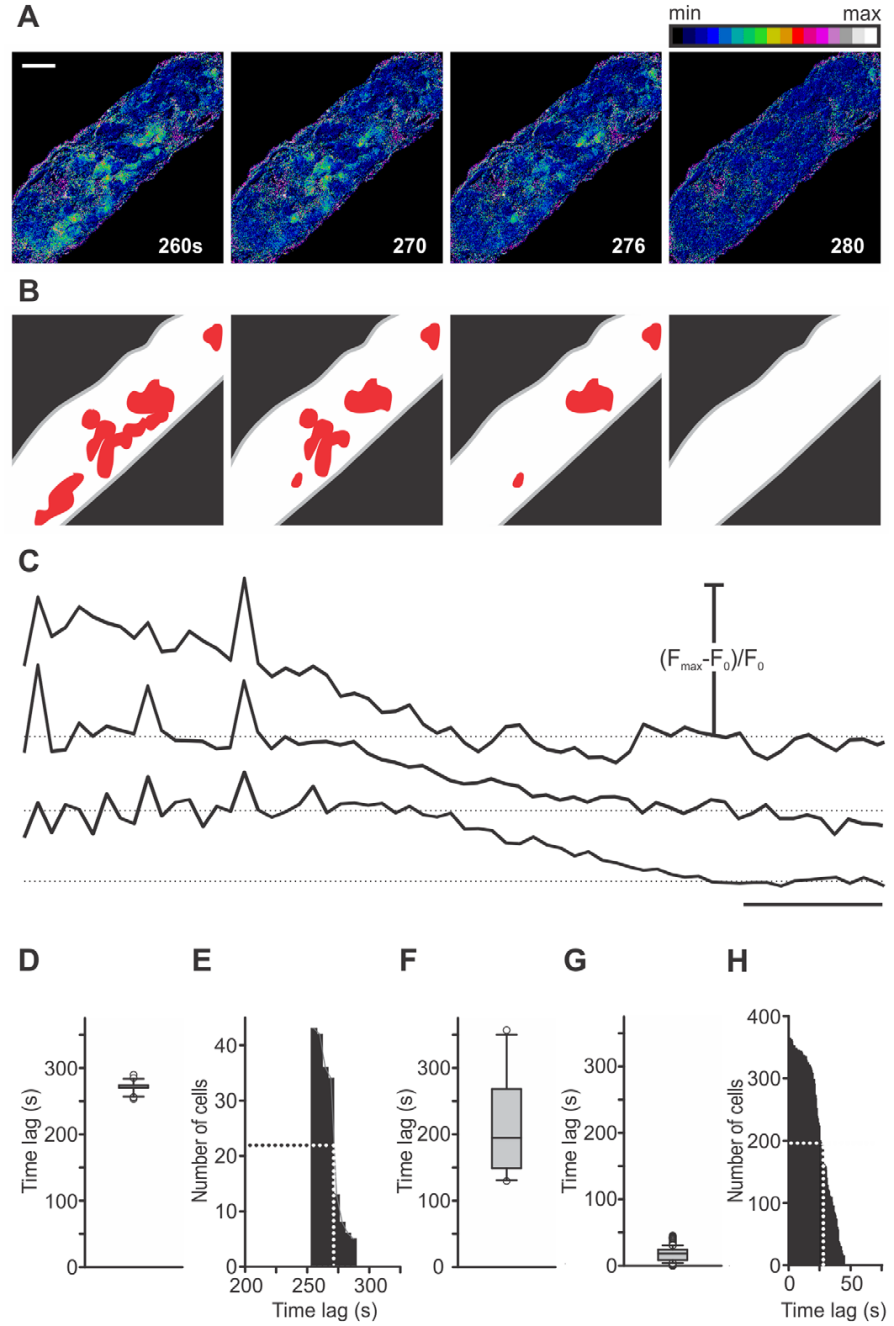
Spatiotemporal characterization of deactivation of beta cells. A Deactivation of beta cells in the islet from Figure 2. Images are presented as F/F_0_ and pseudocolored with blue and white representing low and high intensity signals, respectively. Scale bar indicates 50 µm. **B** Regions that deactivated at a certain time are indicated as red areas that are not present in subsequent images. Differences between individual cells inside these regions were not resolvable at the recording rate of 0.5 Hz. No regular spatial pattern of deactivation is visible. **C** Time traces showing delays in deactivation for the regions indicated in B. Scale bar indicates 20 seconds. On Y-axes, values represent normalized fraction of the difference between maximum and basal fluorescence. **D** Time delays from the end of stimulation to deactivation for 43 cells from this islet (median = 274 s, 1^st^ quartile = 270 s, and 3^rd^ quartile = 274 s). **E** Cumulative distribution of time delays from the end of stimulation to deactivation for 43 cells from this islet. First regions deactivated 256 seconds after lowering glucose to 6 mM. Within the following 36 seconds all other groups of cells deactivated. A half of cells deactivated within 17 seconds after deactivation of the first cell. **F** Distribution of time delays from the end of stimulation to deactivation of the first cell in each islet for 10 different islets (median = 195 s, 1^st^ quartile = 151 s, and 3^rd^ quartile = 261 s). **G** Distribution of time delays from deactivation of the first cell in each islet to deactivation of any given cell from the same islet for 366 cells from 11 different islets (median = 18 s, 1^st^ quartile = 9 s, and 3^rd^ quartile = 24 s). **H** Cumulative distribution of time delays in G. A half of cells deactivated within 22 seconds after the first. All cells deactivated within 45 seconds after the first.

After the glucose had been lowered to basal concentration, beta cells deactivated, a phenomenon unambiguously defined by the return of [Ca^2+^]_i_ from the sustained plateau with superimposed oscillations back to the prestimulatory level. Deactivation was similar to activation in that it did not follow any clearly identifiable spatial pattern but was strikingly different from the activation regarding the time delay after lowering glucose to deactivation of first cells, as well as regarding the degree of synchronicity between individual cells. Figure 6 demonstrates the deactivation process in the same islet of Langerhans as in Figure 2. Similarly as during activation, cells responded to lowering concentration of glucose in groups in which individual responses were not further resolvable at the sampling rate of 0.5 Hz (Figure 6B). First cells deactivated 256 seconds after the solution containing 6 mM glucose had reached the bath chamber (Figure 6D). It is clear that groups of cells were physically separated from each other and located in several different regions of the islet. Similarly as during activation, subsequently deactivated regions did not follow any clearly identifiable spatial pattern. In the particular islet in Figure 6, all cells deactivated within 36 seconds after the first, with a half time of 17 seconds. A more detailed analysis on 366 cells pooled from 10 islets revealed that the median delay from the end of stimulation to deactivation of the first cell in each islet was 195 seconds (1^st^ quartile = 151 s, 3^rd^ quartile = 261 s, Figure 6F) and that the median delay from deactivation of the first cell in each islet to deactivation of any given cell from the same islet was 18 seconds (1^st^ quartile = 9 s, 3^rd^ quartile = 24 s, Figure 6G). On average, by 22 seconds, a half of all cells deactivated (Figure 6H). The cells that deactivated last displayed a time delay of 45 seconds after the first. A comparison with Figure 2 reveals that the time delay from the end of stimulation to deactivation of the first cell in each islet is longer than the time delay from the beginning of stimulation to activation of the first cell in each islet. Further, deactivation is much more synchronous a process than activation, since the time delays between deactivation of the first cell in each islet and deactivation of any given cell from the same islet are shorter than the time delays between activation of the first cell in each islet and activation of any given cell from the same islet.

## Discussion

The tissue-slice technique, coupled with electrophysiological measurements and [Ca^2+^]_i_ imaging techniques has fuelled major advancements in neural and endocrine physiology [28]–[31], [36]–[40]. Since its introduction, the pancreas tissue slice technique has been implemented in electrophysiological experiments [29], [41], [42] and we set out to adapt it to [Ca^2+^]_i_ calcium imaging using CLSM.

We were able to successfully load with the Ca^2+^ fluorescent indicator OGB-1 a large number of cells in cross-sections of islets of Langerhans. In this way we gained access to cells of different types in all layers of islets, surmounting the main shortcoming of imaging techniques involving isolated islets. In our view, this is of great practical importance. In mouse, the relative abundance of a particular cell type is not the same in the fringe compared with the whole islet, with some cell types being over- and others being underrepresented [43]. Considering the importance of paracrine stimuli, the physiological responses of cells in different parts of an islet might vary accordingly. Additionally, individual cells of the same type might differ from each other depending on their localization within the islet [44], [45]. Moreover, not in all species the different cell types are present in the outer layer of the islet. Therefore the slice technique provides an opportunity to study [Ca^2+^]_i_ dynamics in all types of cells in other species [43], [46]. Recording from a large number of cells at a time increases the statistical strength and lowers the number of animals required in experiments, but also increases our chances to conceivably characterize the most rare and only poorly investigated cell types, such as delta-, PP-, or epsilon-cells, irrespective of their position [47]. Finally, with our technique beta cells are becoming accessible to diagnostic tools from complex network theory that could provide us with new ideas on how beta cell syncitia function as a whole [31].

To functionally characterize individual cells we took advantage of earlier work with isolated islets employing [Ca^2+^]_i_ imaging with subsequent immunocytochemical identification, demonstrating that different types of islet cells can be unambiguously recognized by their characteristic [Ca^2+^]_i_ responses to stimulation with glucose [25]–[27], [34]. The boundaries of each cell were clearly discernible due to the lower level of fluorescence at the borders and the inherently variable signal intensities in different cells (See Materials and methods for an outline of possible causes). Since the primary focus of our study was a fundamental spatiotemporal characterization of beta cells, we used a single elementary stimulation protocol, employing 6 mM glucose for the basal conditions, and 12 mM glucose as the stimulus to activate beta cells. A note is in place as to why in contrast to previous work in slices and isolated islets, 6 mM glucose was chosen. First, this concentration lies just below the threshold for beta cell responses in NMRI mice used in this study. We wanted to keep islets at a basal concentration in order to exclude the possibility that after preincubation at stimulatory glucose, islets might develop a glucose memory effect and thus show significantly different responses. Additionally, it has been shown that culture in basal glucose is associated with a slower disappearance of slow as well as fast [Ca^2+^]_i_ oscillations [8]. It was also reported that incubation in lower concentration of glucose can affect basal [Ca^2+^]_i_ levels in islets and their subsequent response to stimuli [8], [15], [48]. Therefore, we chose 6 mM as the highest concentration at which beta cells displayed a constant basal [Ca^2+^]_i_. Noteworthy, in islets in tissue slices as well as in isolated islets basal insulin secretion was observed even at concentrations of glucose lower than 6 mM [7], [28], [49], [50].

As expected, our protocol allowed for detection of beta cells (responses of types 1, 2, and probably 3, Figure 1C–E), and delta cells (type 4 responses, Figure 1F). However, alpha-cells (type 5 responses, Figure 1G) were found only exceptionally, most likely due to the fact that [Ca^2+^]_i_ oscillations subside in the majority of these cells already at glucose concentrations lower than 6 mM. Other protocols, with lowering glucose to levels below 6 mM, shall be tried in future studies to identify alpha-cells. A relative lack of alpha cells explains the marginally above expected percentage of beta- and delta-cells. The cells whose frequencies were not altered by our protocol are most likely already maximally activated delta cells or possibly less glucose-sensitive alpha cells or alpha cells, activated via paracrine stimuli despite increasing glucose. The predominance of beta cells in the core portion of the islet and of the other two cell types in the periphery is in good agreement with previous work [26], [43]. Notably, in a significant number of cells, no clear [Ca^2+^]_i_ signal was present. The already silenced alpha cells probably account for a subset of these cells, and the cells that did not take up the dye for another. However, at least some of the cells were likely rendered unresponsive due to the damage experienced during preparation. Since beta cells are expected to function as a syncitium, the question shall be raised whether the fact that with our procedure a large portion of islet is cut away and that some beta cells in the remaining part could also be damaged, critically alters the responses of individual cells and of the islet as a whole. We consider this highly unlikely. Namely, all beta cells that responded showed synchronized [Ca^2+^]_i_ oscillations. From this and from the fact that for synchronization to occur, electrical coupling is necessary, it is highly unlikely that the cutting procedure critically disrupted the functional syncitium. Most convincingly, the responses of beta cells were not only well synchronized but also strikingly similar in terms of frequencies of [Ca^2+^]_i_ oscillations to what has been obtained with isolated islets studied shortly after isolation and in recordings *in vivo*. In particular, the measured interval between [Ca^2+^]_i_ oscillations (12 seconds) is in the upper range of frequencies determined in isolated islets and in islets *in vivo*. It has been suggested that normal paracrine relationships are retained and the level of important intracellular signaling molecules is less disrupted in fresh preparations and that this could account for the higher frequencies [12], [26]. Further, in our preparation only a negligible fraction of cells (type 3 responses) showed slow [Ca^2+^]_i_ oscillations without a clear plateau and with only some or none superimposed [Ca^2+^]_i_ oscillations. The presence of fast oscillations has been shown to depend on cyclic adenosine monophosphate (cAMP) and it was proposed that their gradual disappearance in culture is a consequence of loss of alpha cells and endogenous glucagon that normally conveys a paracrine cAMP-sustaining tonus to beta cells [8], [12]. In mice, where alpha cells are present predominantly in the periphery of the islets, the isolation procedure involving enzymatic digestion and mechanical trauma is a possible source of damage to alpha cells and disappearance of cAMP-dependent fast oscillations. Since we only exceptionally observed slow [Ca^2+^]_i_ oscillations, we hypothesize that an important loss of paracrine tone does not occur with our preparation and that our work further supports the possibility that slow oscillations could be an experimental epiphenomenon.

[Ca^2+^]_i_ waves repeatedly spreading in an orderly manner across the islet provide evidence that also in normal islets, [Ca^2+^]_i_ waves are the mechanistic substrate for synchronization of [Ca^2+^]_i_ oscillations. The measured speed of [Ca^2+^]_i_ waves in our study (80 µm s^−1^) is in excellent agreement with previous reports [21], [23], [51].

Additionally, our findings shed new light on function of beta cells. Coupling of cells has long been believed to play a crucial role in aligning the responsiveness of a heterogeneous population of beta cells. We confirmed that this is the case. The heterogeneity of cells was clearly visible from differences in time required for activation, but once a beta cell or a group of cells had been activated, all other cells followed and synchronized eventually. Scrutinizing activation and deactivation before and after the sustained plateau where beta cells are well synchronized, we found that the activation process requires almost two orders of magnitude longer time interval (≈100 seconds) to activate beta cells than the synchronization during the plateau phase (≈1 second). Deactivation, while being much more synchronous than activation, still requires at least an order of magnitude longer time interval (≈10 seconds) than the synchronization of [Ca^2+^]_i_ oscillations during the plateau. So why does a cell invariably respond to a relatively small increase or decrease in [Ca^2+^]_i_ in its neighbor by promptly producing or terminating a [Ca^2+^]_i_ oscillation during the plateau phase, while tolerating huge increases in [Ca^2+^]_i_ in its neighbor during activation and deactivation and responding with a delay, one or two orders of magnitude larger? In addition to Ca^2+^ that might well suffice to align the heterogeneity of cells during plateau, something else seems necessary during activation and deactivation. The very magnitude of time delays and the hint of cooperativity that comes from the cumulative distributions point to metabolic activation and possibly to a gradually increasing efficiency of tonically spreading de- and hyperpolarizing electrical signals. The latter might be due to a decrease in membrane conductance or an increase in gap junctional conductance. After a critical level of metabolically driven decrease in membrane permeability (due to closure of the K_ATP_ channels) is reached or gap junctional coupling is increased, the beta cell network can gradually start functioning as a network. Once the whole syncitium is well coupled, hyperpolarizing stimuli due to removal of glucose are expected to be communicated throughout the network much more efficiently than depolarizing ones during activation. This is exactly what we observe by comparing the cumulative distributions of time delays during activation and deactivation. The evidence obtained in favor of these ideas is largely circumstantial and further research will be needed to properly address them, as well as to provide clues to some as yet unresolved issues, such as the asymmetry in delays before first responses during activation and deactivation, the relative invariability of frequency and duration of [Ca^2+^]_i_ oscillations during the plateau, and the striking intercellular homogeneity in duration of oscillations. Namely, while the interval between consecutive oscillations is expected to be constant in excitable systems with [Ca^2+^]_i_ waves spreading in roughly defined directions, the duration of individual oscillation can still be largely determined by the repertoire of membrane channels in individual cells. One notable example of such an excitable system is the myocardium. In beta cell network on the other hand, it seems that the group of cells in which the waves originate determine not only the frequency of oscillations but possibly to at least some extent also their duration.

It should be emphasized that the spatiotemporal characteristics of responses were in no way related to spatiotemporal properties of the perifusion system. We experimentally investigated the concentration changes at different points in the bulk of the fluid above the slice as well as in intercellular spaces within the islet by simultaneously imaging the extracellular florescent dye sulforhodamine B and [Ca^2+^]_i_ (Figure S3).

In our view, the stability of intervals between [Ca^2+^]_i_ oscillations and of their durations is of great practical importance. Since some physiological as well as pharmacological agents are expected to influence beta cells by altering either the frequency of [Ca^2+^]_i_ oscillations or their duration, or both, knowing that during continuous stimulation with glucose neither the frequency nor the duration of [Ca^2+^]_i_ oscillations change intrinsically, all effects upon these two parameters can be ascribed to the factor in question only. As repetitive stimulations can be used in our preparation and are suitable for sequential assessment of different concentrations of a substance or of different components in the same islet, it seems prudent to also investigate how frequency, duration of oscillations, and the various time lags change with repetitive stimulations. This is expected to provide further answers regarding the normal functioning and to produce a framework with which to compare the effects of various physiological and pharmacological substances.

### Conclusions

We have reproducibly introduced a new experimental approach and provided evidence that our *in situ* technique can not only help fill some conceptual gaps between *in vitro* and *in vivo* emerging from work in previous models, but also presented advantages that firmly establish it in the repertoire of techniques from which in the future researchers will choose the best one or the combination of most suitable ones to answer their experimental questions.

## Supporting Information

Figure S1 **Viability of cells in islets of Langerhans in tissue slices.** Viability of cells at the depth at which recordings of [Ca^2+^]_i_ were performed was checked employing the commercially available two-color fluorescence LIVE/DEAD® Viability/Cytotoxicity Kit (Invitrogen, Eugene, Oregon, USA) that is based on two probes measuring intracellular esterase activity and integrity of the plasma membrane, which are recognized parameters of cell viability. Live cells (green) are labeled with component A of the kit (calcein acetoxymethlyester), since intracellular esterase activity is needed for the presence of the dye in the cytosol. The component B (ethidium homodimer-1) binds to nuclei of dead cells (red) when plasma membrane integrity is lost and enables entry of component B into the cell. Slicing of the agarose-embedded pancreatic islet resulted in damage to the superficial layer of cells. In deeper layers, starting at 2–3 cells below the cutting surface (i.e. 15–20 microns below the surface) mostly calcein labeled cells were detected, with only a few ethidium labeled cells, indicating that a great majority of cells were viable at the optical section from which the recordings of the [Ca^2+^]_i_ were made. Yellow lines indicate the position of the image within the Z-stack. Depth of the Z-stack was 45 µm. Scale bar indicates 50 µm.
(TIF)

Figure S2 **Morphology of cells in islets of Langerhans in tissue slices.** To assess cell morphology in our preparation, we labeled the membranes of cells within an islet of Langerhans with the lipophilic dye di-4-ANNEPS. Shown are imaging layers at 10 µm (A), 20 µm (B) and 30 µm (C) below surface of the tissue slice. Note that characteristic polyhedral shapes of cells are well preserved. Scale bar indicates 25 µm.
(TIF)

Figure S3 **Concentration changes in the chamber and in the intercellular space. A** Geometry of the bath chamber used in our experiments. The tissue slice was superfused constantly at a rate of 13 µl/s. The volume of extracellular solution (ECS) in the chamber was 1.5 ml. ECS entered the chamber at one border (Inflow) and exited it at the other (Outflow). Points at which concentration changes were assessed are indicated in black, red, and green. **B** Employing the polar extracellular fluorescent tracer sulforhodamine B (20 mg/l; Invitrogen, Eugene, Oregon, USA), the temporal evolution of fluorescence at points indicated in A was tracked in the bulk ECS just above the islet. At the recording rate of 1 Hz, no clear delays could be detected between onsets of increases in the fluorescence, indicated in colors corresponding to colors of the three points indicated in A. **C** Fluorescence signals recorded from intercellular spaces at the three points indicated in A, at a depth within the islet at which recordings of [Ca^2+^]_i_ were typically performed. The concentration of sulforhodamine B increased slower than in the solution above the slice. However, the temporal evolution at different locations followed practically the same time course. **D** [Ca^2+^]_i_-dependent fluorescence was recorded simultaneously with the fluorescence of the extracellular indicator sulforhodamine B. Cells situated at the points indicated in A activated with clearly pronounced time delays despite virtually identical increases in extracellular fluorescence at these points, practically excluding the possibility that unequal access of glucose to individual cells could be responsible for the time delays between activations of individual cells observed in our study. An effective equilibration with the concentration in the solution above the slice was typically reached by the time when high frequency [Ca^2+^]_i_ oscillations on the sustained elevation of [Ca^2+^]_i_ were established. Scale bar indicates 60 seconds.
(TIF)

Video S1 **Spatiotemporal characterization of activation of beta cells.** Ca^2+^-dependent fluorescence signal in the islet of Langerhans shown in Figure 2, showing activation of beta cells upon stimulation with 12 mM glucose. A total of 70 frames recorded between 90 and 230 seconds after the beginning of stimulation with 12 mM glucose are presented at a rate of 2 frames s^−1^. Individual frames are presented as F/F_0_ and pseudocolored with blue and white representing low and high intensity signals, respectively.
(MP4)

Video S2 **Spatiotemporal characterization of [Ca^2+^]_i_ oscillations superimposed on the sustained plateau.** Ca^2+^-dependent fluorescence signal in the islet of Langerhans shown in Figure 5. A total of 2400 frames recorded over a period of 120 seconds are presented at a rate of 100 frames s^−1^. Individual frames are presented as F/F_0_ and pseudocolored with blue and white representing low and high intensity signals, respectively. [Ca^2+^]_i_ waves repeatedly spread from top left to bottom right. Towards the end of the recording, the [Ca^2+^]_i_ wave did not always spread to the region at the right end of the islet, indicating a variable efficiency of the synchronizing mechanism.
(MP4)

Video S3 **Viability of cells in islets of Langerhans in tissue slices.** A Z-stack of the islet of Langerhans labeled with the LIVE/DEAD® Viability/Cytotoxicity Kit (Invitrogen, Eugene, Oregon, USA) and shown in Figure S1. Live and dead cells shown in green and red, respectively. Indicated is the depth of the optical section with respect to the surface of the tissue slice. Scale bar indicates 50 µm.
(MP4)

## References

[1] Berridge MJ, Lipp P, Bootman MD (2000) The versatility and universality of calcium signalling. Nat Rev Mol Cell Biol 1: 11–21. 11413485 10.1038/35036035

[2] Rorsman P, Braun M, Zhang Q (2012) Regulation of calcium in pancreatic α- and β-cells in health and disease. Cell Calcium 51: 300–308. 22177710 10.1016/j.ceca.2011.11.006 PMC3334273

[3] Grynkiewicz G, Poenie M, Tsien RY (1985) A new generation of Ca2+ indicators with greatly improved fluorescence properties. Journal of Biological Chemistry 260: 3440–3450. 3838314

[4] Tsien RY (1981) A non-disruptive technique for loading calcium buffers and indicators into cells. Nature 290: 527–528. 7219539 10.1038/290527a0

[5] Santos RM, Rosario LM, Nadal A, Garcia-Sancho J, Soria B, et al (1991) Widespread synchronous Ca oscillations due to bursting electrical activity in single pancreatic islets. Pflügers Archiv European Journal of Physiology 418: 417–422. 1876486 10.1007/BF00550880

[6] Gilon P, Henquin JC (1992) Influence of membrane potential changes on cytoplasmic Ca2+ concentration in an electrically excitable cell, the insulin-secreting pancreatic B-cell. Journal of Biological Chemistry 267: 20713–20720. 1400388

[7] Gilon P, Shepherd RM, Henquin JC (1993) Oscillations of secretion driven by oscillations of cytoplasmic Ca^2+^ as evidenced in single pancreatic islets. Journal of Biological Chemistry 268: 22265–22268. 8226733

[8] Bergsten P (1995) Slow and fast oscillations of cytoplasmic Ca2+ in pancreatic islets correspond to pulsatile insulin release. American Journal of Physiology - Endocrinology And Metabolism 268: E282–E287. 10.1152/ajpendo.1995.268.2.E282 7864105

[9] Fernandez J, Valdeolmillos M (2000) Synchronous glucose-dependent [Ca2+]i oscillations in mouse pancreatic islets of Langerhans recorded in vivo. FEBS Letters 477: 33–36. 10899306 10.1016/s0014-5793(00)01631-8

[10] Sánchez-Andrés JV, Gomis A, Valdeolmillos M (1995) The electrical activity of mouse pancreatic beta-cells recorded in vivo shows glucose-dependent oscillations. J Physiol 486: 223–228. 7562637 10.1113/jphysiol.1995.sp020804 PMC1156510

[11] Beauvois MC, Merezak C, Jonas J-C, Ravier MA, Henquin J-C, et al (2006) Glucose-induced mixed [Ca2+]c oscillations in mouse β-cells are controlled by the membrane potential and the SERCA3 Ca2+-ATPase of the endoplasmic reticulum. American Journal of Physiology - Cell Physiology 290: C1503–C1511. 16381799 10.1152/ajpcell.00400.2005

[12] Liu Y-J, Tengholm A, Grapengiesser E, Hellman B, Gylfe E (1998) Origin of slow and fast oscillations of Ca2+ in mouse pancreatic islets. J Physiol 508: 471–481. 9508810 10.1111/j.1469-7793.1998.471bq.x PMC2230881

[13] Valdeolmillos M, Santos RM, Contreras D, Soria B, Rosario LM (1989) Glucose-induced oscillations of intracellular Ca2+ concentration resembling bursting electrical activity in single mouse islets of Langerhans. FEBS Letters 259: 19–23. 2689228 10.1016/0014-5793(89)81484-x

[14] Bergsten P, Grapengiesser E, Gylfe E, Tengholm A, Hellman B (1994) Synchronous oscillations of cytoplasmic Ca2+ and insulin release in glucose-stimulated pancreatic islets. Journal of Biological Chemistry 269: 8749–8753. 8132606

[15] Gilon P, Jonas J, Henquin J (1994) Culture duration and conditions affect the oscillations of cytoplasmic calcium concentration induced by glucose in mouse pancreatic islets. Diabetologia 37: 1007–1014. 7851679 10.1007/BF00400464

[16] Grapengiesser E, Gylfe E, Hellman B (1988) Glucose-induced oscillations of cytoplasmic Ca2+ in the pancreatic β-cell. Biochemical and Biophysical Research Communications 151: 1299–1304. 3281672 10.1016/s0006-291x(88)80503-5

[17] Valdeolmillos M, Gomis A, Sánchez-Andrés JV (1996) In vivo synchronous membrane potential oscillations in mouse pancreatic beta-cells: lack of co-ordination between islets. J Physiol 493: 9–18. 8735691 10.1113/jphysiol.1996.sp021361 PMC1158947

[18] Meissner HP (1976) Electrophysiological evidence for coupling between [beta] cells of pancreatic islets. Nature 262: 502–504. 785280 10.1038/262502a0

[19] Eddlestone GT, Gonçalves A, Bangham JA, Rojas E (1984) Electrical coupling between cells in islets of langerhans from mouse. Journal of Membrane Biology 77: 1–14. 6321740 10.1007/BF01871095

[20] Palti Y, BenDavid G, Lachov E, Mika YH, Omri G, et al (1996) Islets of Langerhans generate wavelike electric activity modulated by glucose concentration. Diabetes 45: 595–601. 8621009 10.2337/diab.45.5.595

[21] Benninger RK, Zhang M, Head WS, Satin LS, Piston DW (2008) Gap junction coupling and calcium waves in the pancreatic islet. Biophys J 95: 5048–5061. 18805925 10.1529/biophysj.108.140863 PMC2586567

[22] Bertuzzi F, Davalli AM, Nano R, Socci C, Codazzi F, et al (1999) Mechanisms of coordination of Ca^2+^ signals in pancreatic islet cells. Diabetes 48: 1971–1978. 10512361 10.2337/diabetes.48.10.1971

[23] Zhang Q, Galvanovskis J, Abdulkader F, Partridge CJ, Gopel SO, et al (2008) Cell coupling in mouse pancreatic beta-cells measured in intact islets of Langerhans. Philos Transact A Math Phys Eng Sci 366: 3503–3523. 10.1098/rsta.2008.0110 18632454

[24] Ravier MR, Sehlin JS, Henquin JH (2002) Disorganization of cytoplasmic Ca^2+^ oscillations and pulsatile insulin secretion in islets from *ob/ob* mice. Diabetologia 45: 1154–1163. 12189446 10.1007/s00125-002-0883-9

[25] Asada N, Shibuya I, Iwanaga T, Niwa K, Kanno T (1998) Identification of alpha- and beta-cells in intact isolated islets of Langerhans by their characteristic cytoplasmic Ca^2+^ concentration dynamics and immunocytochemical staining. Diabetes 47: 751–757. 9588446 10.2337/diabetes.47.5.751

[26] Nadal A, Quesada I, Soria B (1999) Homologous and heterologous asynchronicity between identified α-, β- and δ-cells within intact islets of Langerhans in the mouse. J Physiol 517: 85–93. 10226151 10.1111/j.1469-7793.1999.0085z.x PMC2269319

[27] Quesada I, Todorova MG, Alonso-Magdalena P, Beltrá M, Carneiro EM, et al (2006) Glucose Induces Opposite Intracellular Ca^2+^ Concentration Oscillatory Patterns in Identified α- and β-Cells Within Intact Human Islets of Langerhans. Diabetes 55: 2463–2469. 16936194 10.2337/db06-0272

[28] Speier S, Rupnik M (2003) A novel approach to in situ characterization of pancreatic ß-cells. Pflügers Archiv European Journal of Physiology 446: 553–558. 12774232 10.1007/s00424-003-1097-9

[29] Rupnik M (2009) The physiology of rodent beta-cells in pancreas slices. Acta Physiologica 195: 123–138. 18983446 10.1111/j.1748-1716.2008.01927.x

[30] Sánchez-Cárdenas C, Hernández-Cruz A (2010) GnRH-Induced Ca^2+^-Signalling Patterns in Mouse Gonadotrophs Recorded from Acute Pituitary Slices in vitro. Neuroendocrinology 91: 239–255. 20090289 10.1159/000274493

[31] Hodson DJ, Schaeffer M, Romano N, Fontanaud P, Lafont C, et al (2012) Existence of long-lasting experience-dependent plasticity in endocrine cell networks. Nat Commun 3: 605. 22215080 10.1038/ncomms1612 PMC3272579

[32] Poole CA, Brookes NH, Clover GM (1993) Keratocyte networks visualised in the living cornea using vital dyes. Journal of Cell Science 106: 685–691. 8282773 10.1242/jcs.106.2.685

[33] Takahashi N, Nemoto T, Kimura R, Tachikawa A, Miwa A, et al (2002) Two-Photon Excitation Imaging of Pancreatic Islets With Various Fluorescent Probes. Diabetes 51: S25–S28. 11815453 10.2337/diabetes.51.2007.s25

[34] Quesada I, Nadal A, Soria B (1999) Different effects of tolbutamide and diazoxide in alpha, beta-, and delta-cells within intact islets of Langerhans. Diabetes 48: 2390–2397. 10580428 10.2337/diabetes.48.12.2390

[35] Milton F (1937) The Use of Ranks to Avoid the Assumption of Normality Implicit in the Analysis of Variance. Journal of the American Statistical Association 32: 675–701.

[36] Schneggenburger R, Forsythe I (2006) The calyx of Held. Cell and Tissue Research 326: 311–337. 16896951 10.1007/s00441-006-0272-7

[37] Moser T, Neher E (1997) Rapid Exocytosis in Single Chromaffin Cells Recorded from Mouse Adrenal Slices. The Journal of Neuroscience 17: 2314–2323. 9065492 10.1523/JNEUROSCI.17-07-02314.1997 PMC6573505

[38] Edwards FA, Konnerth A, Sakmann B, Takahashi T (1989) A thin slice preparation for patch clamp recordings from neurones of the mammalian central nervous system. Pflügers Archiv European Journal of Physiology 414: 600–612. 2780225 10.1007/BF00580998

[39] García AG, García-De-Diego AM, Gandía L, Borges R, García-Sancho J (2006) Calcium Signaling and Exocytosis in Adrenal Chromaffin Cells. Physiological Reviews 86: 1093–1131. 17015485 10.1152/physrev.00039.2005

[40] Sedej S, Rose T, Rupnik M (2005) cAMP increases Ca^2+^-dependent exocytosis through both PKA and Epac2 in mouse melanotrophs from pituitary tissue slices. J Physiol 567: 799–813. 15994184 10.1113/jphysiol.2005.090381 PMC1474225

[41] Huang Y-C, Rupnik M, Gaisano HY (2011) Unperturbed islet α-cell function examined in mouse pancreas tissue slices. J Physiol 589: 395–408. 21078586 10.1113/jphysiol.2010.200345 PMC3043540

[42] Speier S, Gjinovci A, Charollais A, Meda P, Rupnik M (2007) Cx36-Mediated Coupling Reduces β-Cell Heterogeneity, Confines the Stimulating Glucose Concentration Range, and Affects Insulin Release Kinetics. Diabetes 56: 1078–1086. 17395748 10.2337/db06-0232

[43] Cabrera O, Berman DM, Kenyon NS, Ricordi C, Berggren P-O, et al (2006) The unique cytoarchitecture of human pancreatic islets has implications for islet cell function. Proc Natl Acad Sci U S A 103: 2334–2339. 16461897 10.1073/pnas.0510790103 PMC1413730

[44] Katsuta H, Akashi T, Katsuta R, Nagaya M, Kim D, et al (2010) Single pancreatic beta cells co-express multiple islet hormone genes in mice. Diabetologia 53: 128–138. 19851748 10.1007/s00125-009-1570-x PMC2789931

[45] Bosco D, Rouiller DG, Halban PA (2007) Differential expression of E-cadherin at the surface of rat β-cells as a marker of functional heterogeneity. Journal of Endocrinology 194: 21–29. 17592017 10.1677/JOE-06-0169

[46] Heller RS (2010) The Comparative Anatomy of Islets. In: Islam MS, editor. Islets of Langerhans. Berlin: Springer-Verlag Berlin. 21–37. 10.1007/978-90-481-3271-3_2 20217492

[47] Prado CL, Pugh-Bernard AE, Elghazi L, Sosa-Pineda B, Sussel L (2004) Ghrelin cells replace insulin-producing β cells in two mouse models of pancreas development. Proc Natl Acad Sci U S A 101: 2924–2929. 14970313 10.1073/pnas.0308604100 PMC365721

[48] Hellman B, Gylfe E, Grapengiesser E, Lund P-E, Berts A (1992) Cytoplasmic Ca^2+^ oscillations in pancreatic ß-cells. Biochimica et Biophysica Acta (BBA) - Reviews on Biomembranes 1113: 295–305. 1450203 10.1016/0304-4157(92)90003-s

[49] Rozzo A, Meneghel-Rozzo T, Delakorda SL, Yang S-B, Rupnik M (2009) Exocytosis of Insulin. Annals of the New York Academy of Sciences 1152: 53–62. 19161376 10.1111/j.1749-6632.2008.04003.x

[50] Bergsten P, Hellman B (1993) Glucose-Induced Cycles of Insulin Release Can Be Resolved into Distinct Periods of Secretory Activity. Biochemical and Biophysical Research Communications 192: 1182–1188. 8507191 10.1006/bbrc.1993.1541

[51] Aslanidi OV, Mornev OA, Skyggebjerg O, Arkhammar P, Thastrup O, et al (2001) Excitation Wave Propagation as a Possible Mechanism for Signal Transmission in Pancreatic Islets of Langerhans. Biophys J 80: 1195–1209. 11222284 10.1016/S0006-3495(01)76096-1 PMC1301315

