# Supplementary figures and images for: Glucose-Stimulated Calcium Dynamics in Islets of Langerhans in Acute Mouse Pancreas Tissue Slices
Source: PLoS One. 2013 Jan 24;8(1):e54638. doi: 10.1371/journal.pone.0054638 (PMC3554663; doi:10.1371/journal.pone.0054638)

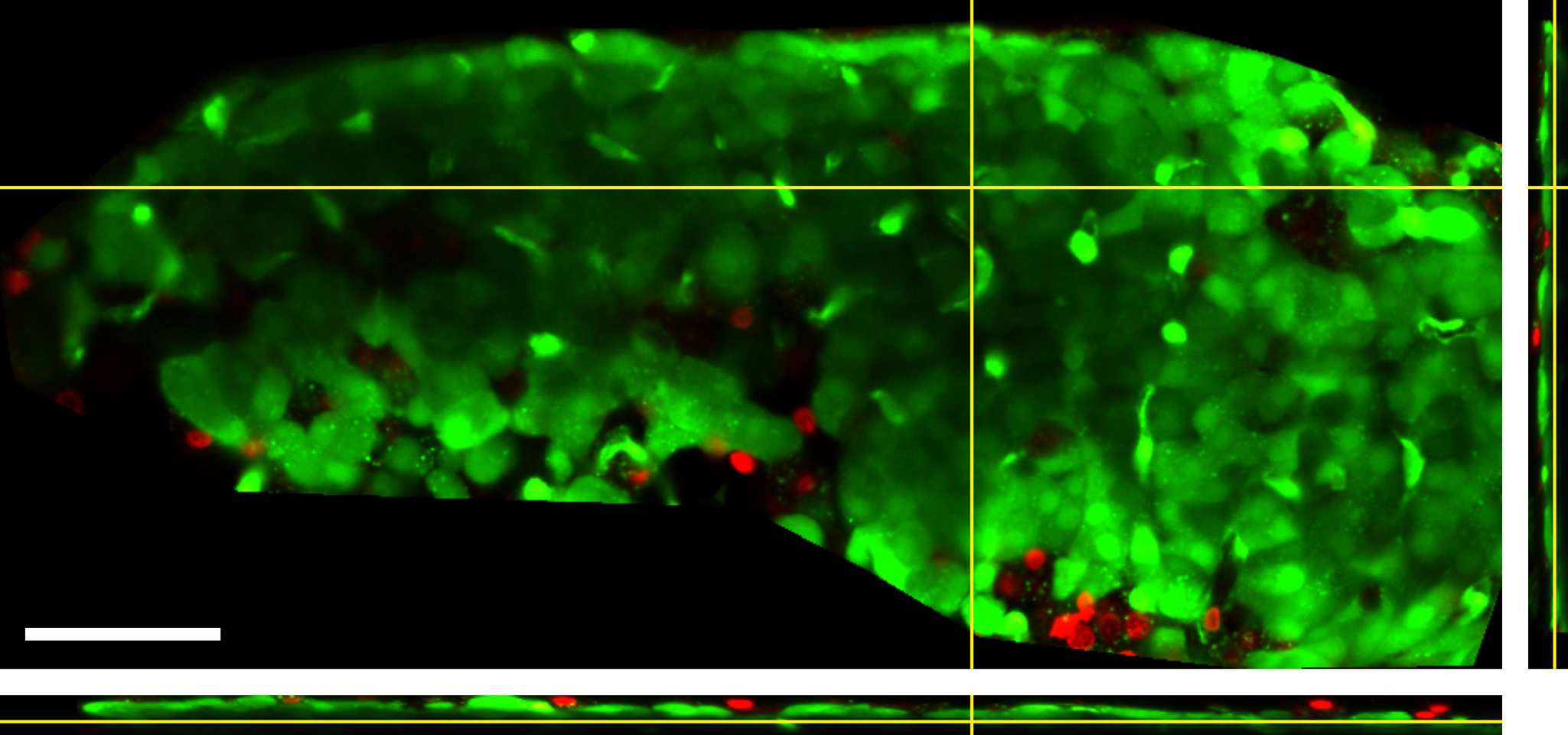

Supplement: Figure S1 — Viability of cells in islets of Langerhans in tissue slices. Viability of cells at the depth at which recordings of [Ca2+]i were performed was checked employing the commercially available two-color fluorescence LIVE/DEAD® Viability/Cytotoxicity Kit (Invitrogen, Eugene, Oregon, USA) that is based on two probes measuring intracellular esterase activity and integrity of the plasma membrane, which are recognized parameters of cell viability. Live cells (green) are labeled with component A of the kit (calcein acetoxymethlyester), since intracellular esterase activity is needed for the presence of the dye in the cytosol. The component B (ethidium homodimer-1) binds to nuclei of dead cells (red) when plasma membrane integrity is lost and enables entry of component B into the cell. Slicing of the agarose-embedded pancreatic islet resulted in damage to the superficial layer of cells. In deeper layers, starting at 2–3 cells below the cutting surface (i.e. 15–20 microns below the surface) mostly calcein labeled cells were detected, with only a few ethidium labeled cells, indicating that a great majority of cells were viable at the optical section from which the recordings of the [Ca2+]i were made. Yellow lines indicate the position of the image within the Z-stack. Depth of the Z-stack was 45 µm. Scale bar indicates 50 µm. (TIF) [file pone.0054638.s001.tif]

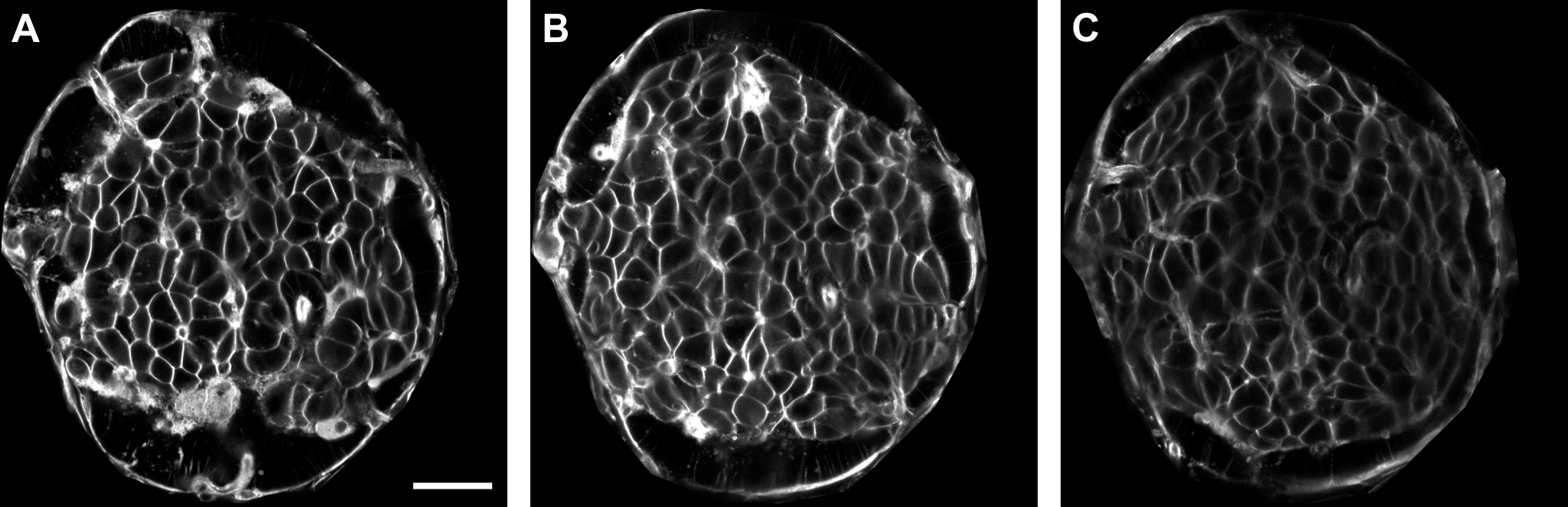

Supplement: Figure S2 — Morphology of cells in islets of Langerhans in tissue slices. To assess cell morphology in our preparation, we labeled the membranes of cells within an islet of Langerhans with the lipophilic dye di-4-ANNEPS. Shown are imaging layers at 10 µm (A), 20 µm (B) and 30 µm (C) below surface of the tissue slice. Note that characteristic polyhedral shapes of cells are well preserved. Scale bar indicates 25 µm. (TIF) [file pone.0054638.s002.tif]

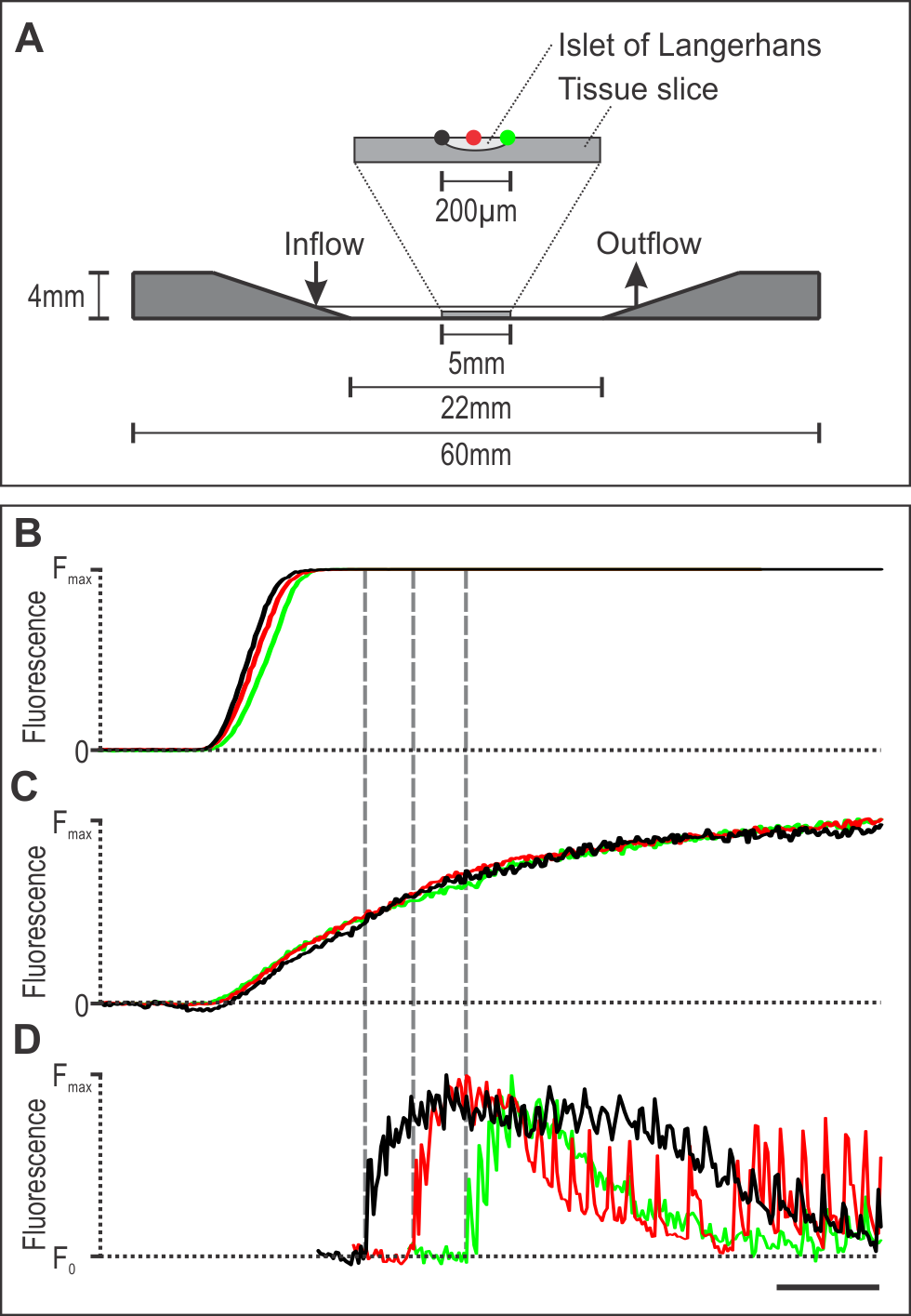

Supplement: Figure S3 — Concentration changes in the chamber and in the intercellular space. A Geometry of the bath chamber used in our experiments. The tissue slice was superfused constantly at a rate of 13 µl/s. The volume of extracellular solution (ECS) in the chamber was 1.5 ml. ECS entered the chamber at one border (Inflow) and exited it at the other (Outflow). Points at which concentration changes were assessed are indicated in black, red, and green. B Employing the polar extracellular fluorescent tracer sulforhodamine B (20 mg/l; Invitrogen, Eugene, Oregon, USA), the temporal evolution of fluorescence at points indicated in A was tracked in the bulk ECS just above the islet. At the recording rate of 1 Hz, no clear delays could be detected between onsets of increases in the fluorescence, indicated in colors corresponding to colors of the three points indicated in A. C Fluorescence signals recorded from intercellular spaces at the three points indicated in A, at a depth within the islet at which recordings of [Ca2+]i were typically performed. The concentration of sulforhodamine B increased slower than in the solution above the slice. However, the temporal evolution at different locations followed practically the same time course. D [Ca2+]i-dependent fluorescence was recorded simultaneously with the fluorescence of the extracellular indicator sulforhodamine B. Cells situated at the points indicated in A activated with clearly pronounced time delays despite virtually identical increases in extracellular fluorescence at these points, practically excluding the possibility that unequal access of glucose to individual cells could be responsible for the time delays between activations of individual cells observed in our study. An effective equilibration with the concentration in the solution above the slice was typically reached by the time when high frequency [Ca2+]i oscillations on the sustained elevation of [Ca2+]i were established. Scale bar indicates 60 seconds. (TIF) [file pone.0054638.s003.tif]
